# The two-year impact of first generation protease inhibitor based antiretroviral therapy (PI-ART) on health-related quality of life

**DOI:** 10.1186/1477-7525-3-32

**Published:** 2005-05-04

**Authors:** Lars E Eriksson, Göran A Bratt, Eric Sandström, Gun Nordström

**Affiliations:** 1 Department of Nursing, Karolinska Institutet, 23300, SE-141 83 Huddinge, Sweden; 2 Department of Venhälsan, South Stockholm General Hospital, SE-118 83 Stockholm, Sweden; 3 Department of Virology, Swedish Institute for Infectious Disease Control, SE-171 82 Solna, Sweden

## Abstract

**Background:**

Protease inhibitor based antiretroviral therapy (PI-ART) was introduced in 1996 and has greatly reduced the incidence of HIV-related morbidity and mortality in the industrialised world. PI-ART would thus be expected to have a positive effect on health-related quality of life (HRQL). On the other hand, HRQL might be negatively affected by strict adherence requirements as well as by short and long-term adverse effects. The aim of this study was to assess the influence of two years of first generation PI-ART on HRQL in patients with a relatively advanced state of HIV-infection. Furthermore, we wanted to investigate the relation between developments in HRQL and viral response, self-reported adherence and subjective experience of adverse effects in patients with PI-ART.

**Methods:**

HRQL was measured by the Swedish Health-Related Quality of Life Questionnaire (SWED-QUAL). Sixty-three items from the SWED-QUAL forms two single-item and 11 multi-item dimension scales. For this study, two summary SWED-QUAL scores (physical HRQL composite score and emotional HRQL composite score) were created through a data reduction procedure. At the 2-year follow-up measurement (see below), items were added to measure adherence and subjective experience of adverse effects. Demographic and medical data were obtained from specific items in the questionnaires and from the medical files. Seventy-two patients who were among the first to receive PI-ART (indinavir or ritonavir based) responded to the questionnaire before the start of PI-ART. Of these, 54 responded to the same instrument after two years of treatment (13 had died, four had changed clinic and one did not receive the questionnaire).

**Results:**

The main findings were that the emotional HRQL deteriorated during two years of PI-ART, while the physical HRQL remained stable. Multiple linear regression analyses showed that experience of adverse effects contributed most to the deterioration of emotional HRQL.

**Conclusion:**

In this sample of patients with relatively advanced state of HIV-infection, our data suggested that a negative development of physical HRQL had been interrupted by the treatment and that the emotional dimension of HRQL deteriorated during two years after start of PI-ART. Subjective experience of adverse effects made a major contribution to the decrease in emotional HRQL. The results underline the importance of including HRQL measures in the evaluation of new life prolonging therapies.

## Background

Protease inhibitor based antiretroviral therapy (PI-ART), defined as the combination of at least two nucleoside analogues with at least one protease inhibitor (PI) [1], was introduced in 1996 and has greatly reduced the incidence of HIV-related morbidity and mortality in the industrialised world [2,3]. PI-ART would thus be expected to have a positive effect on health-related quality of life (HRQL). On the other hand, HRQL might be negatively affected by strict adherence requirements as well as by short and long-term adverse effects [4,5].

Several studies (cross-sectional or longitudinal) have focused on the HRQL of HIV-positive individuals in different stages of the HIV infection and under different treatment regimes. The results have varied but in general HIV infection affects several physical, psychological and social dimensions of HRQL and patients with symptomatic disease and/or an AIDS-defining complication are more severely affected than those with other comparable chronic diseases [6-8]. HRQL has been shown to be related to the CD4 value, viral load and symptoms, so that patients with a more advanced state of HIV infection reported poorer HRQL [9-15]. Moreover, symptoms, physical function, role function and sexual function deteriorated over time, while emotional domains were unchanged or improved [9,10,16]. Only a few studies on the influence of long-term (>1 year) PI-ART on the quality of life have yet been published. Nieuwkerk *et al*. [17] compared the development of HRQL during three different PI based regimes and concluded that in terms of HRQL, patients with higher CD4 values at start experienced less benefit from the treatment. Burgoyne *et al. *[18,19] followed HRQL in 41 patients with different treatment status over a period of four years and found no overall change of HRQL and that HRQL was less sensitive to CD4 changes than to symptom changes as well as that change in HRQL was somewhat related to change in social support.

The main aim of the present study was to investigate HRQL before and after two years of first generation PI-ART. The following research questions were addressed: (a) Does HRQL change during two years of PI-ART? (b) Do viral response, adherence, subjective experience of adverse effects and initial CD4 count predict changes in HRQL during two years of PI-ART?

## Methods

### Data collection

#### Subjects

The study was performed at the Department of Venhälsan at South Stockholm General Hospital, Sweden. A convenient sample of 72 subjects (70 men and 2 women) in an advanced state of HIV infection and who were among the first patients to receive PI-ART in Sweden responded to the HRQL instrument described below before the start of PI-ART (*pre PI-ART*). HIV infection was documented by at least two laboratory tests (two repeated ELISA tests or one ELISA test and one Western Blot). The patients were treated according to best clinical practice and the participant's physician chose the drug combination of the PI-ART (at least 2 nucleoside analogue reverse transcriptase inhibitors and either indinavir (n = 55) or ritonavir (n = 17) at start). Approximately two years after the introduction of PI-ART (*follow-up*; mean 25.1 months, standard deviation (SD) 2.8 months, post initiation), 54 of the 72 subjects (75 %) completed the *follow-up *measure. Thirteen patients had died, four had changed clinic and one did not receive the *follow-up *questionnaire.

#### The Swedish Health-Related Quality of Life Questionnaire (SWED-QUAL)

The patients completed the SWED-QUAL at the *pre PI-ART *and two-year *follow-up *visits. SWED-QUAL was developed by Brorsson *et al. *[20] from the measures used in the US Medical Outcomes Study (MOS) [21-24]. The questionnaire, which is designed to measure HRQL, consists of 70 items, of which 63 forms two single-item and 11 multi-item dimension scales of Likert type: physical functioning (7 items), mobility (1 item), satisfaction with physical ability (1 item), role limitations due to physical health (3 items), pain (6 items), emotional well-being: positive affect (i.e. positive feelings; 6 items), emotional well-being: negative affect (i.e. negative feelings; 6 items), role limitations due to emotional health (3 items), sleep problems (7 items), satisfaction with family life (relations with parents, siblings, children etc.; 4 items), relation to partner (6 items), sexual functioning (4 items) and general health perception (9 items). In the present study, the relation to partner section was slightly modified to make it suitable for the investigated group (i.e. the word "spouse" was replaced by "partner"). Each scale is transformed into a 0–100 index; the higher the score, the better the perceived HRQL. In a general population sample, the internal-consistency reliability coefficients (Cronbach's α) ranged from 0.79 to 0.89 [20]. Brorsson *et al. *have also reportedpreliminary support for the construct validity [20]. The instrument has been used in our previous study of HIV-positive subjects in Sweden [25].

A factor analysis (see below) was performed on a merged material from three groups of patients: (a) the group described above at *pre PI-ART *(n = 72), (b) a previously described group of protease inhibitor naïve HIV patients (n = 73) [25] and (c) an unpublished material of 164 HIV-negative men who have sex with men visiting our information and screening clinic for sexually transmitted diseases (own unpublished data).

#### Adherence/adverse effects

At the *follow-up*, the patients' adherence and drug-related adverse effects were examined. Adherence was assessed by asking the following question, modified from Morisky *et al. *[26]: "How many times during the last month did you skip doses of your HIV medication for the following reasons: because you felt so good that it did not matter, because you felt worse due to the medication or because you forgot?" For each of the three given reasons the patients were asked to rate the number of missed doses on a scale with five alternatives (1 = 0 doses, 2 = 1–2 doses, 3 = 3–5 doses, 4 = 6–9 doses or 5 = >9 doses). Patients were classified as non-adherent if they indicated >9 missed doses in at least one of the three mentioned categories (i.e. indicating 90% adherence or less) or if they indicated missed doses in all three categories.

The subjective experience of medication-related adverse effects was addressed in one question, which asked the patients to rate their global experience of adverse effects during the past month on a 10 cm visual analogue scale (VAS) ranging from "none at all" to "severe".

#### Laboratory measures

Lymphocyte subsets were determined, using routine flow cytometry [27], at least every 3–4 months. The level of HIV-1 RNA in plasma was quantified at the Swedish Institute for Infectious Disease Control by a commercially available reverse transcriptase polymerase chain reaction (PCR)-based assay (HIV Monitor; Roche Diagnostic Systems, Branchburg New Jersey, USA). The level of quantification at the time of the study was 500 viral copies ml^-1^.

#### Viral outcome

On the basis of the participants' long-term virologic outcome to PI-ART, they were subdivided into viral responders and viral non-responders. Viral responders were defined as participants who, after the first 3 months of PI-ART, had either HIV RNA less than 500 copies ml^-1 ^in more than 75% of the analysed samples or a continuous decrease in HIV RNA to below 500 copies ml^-1 ^before 18 months. All other participants were regarded as viral non-responders [28].

#### Demographic and medical data

Demographic and medical data were obtained from specific items in the questionnaires and from the medical files.

### Statistical methods

Statistical calculations were performed with the assistance of the personal-computer program SPSS for Windows, version 11.0.0. The criterion for statistical significance was *p *< 0.05. Since some parameters did not fulfil the assumptions of a normal distribution, the Wilcoxon signed ranks test [29] was used to compare differences between two related groups (i.e. *pre PI-ART *versus *follow-up*).

In order to achieve data reduction to create composite scores for use in multiple regression analyses of the material, a rotated component matrix analysis [30] was performed on the scores from ten of the 13 SWED-QUAL scales (i.e. those measuring physical and emotional health). The following scales were used in the factor analysis: physical functioning, mobility, satisfaction with physical ability, role limitations due to physical health, pain, emotional well-being: positive affect, emotional well-being: negative affect, role limitations due to emotional health, sleep problems and sexual functioning. The analysis resulted in two factors: (a) physical HRQL composite score, i.e. PCS, comprising the averaged scores of the SWED-QUAL scales physical functioning, mobility, satisfaction with physical ability, pain, role limitations due to physical health and sexual functioning, and (b) emotional HRQL composite score, i.e. ECS, comprising the averaged scores of the SWED-QUAL scales role limitations due to emotional health, positive affect, negative affect and sleep problems (Table 1). These two factors explained 65 % of the total variance.

**Table 1:** Rotated factor loading (varimax) of the principal component analysis (n = 309)

	**Rotated factor loadings**	
	
	**PCS**	**ECS**
**SWED-QUAL Scale**		
Physical functioning	0.92	0.07
Mobility	0.83	0.04
Satisfaction with physical ability	0.79	0.24
Pain	0.56	0.45
Role limitations due to physical health	0.66	0.46
Sexual functioning	0.63	0.32
Role limitations due to emotional health	0.33	0.75
Emotional well-being, positive affect	0.08	0.81
Emotional well-being, negative affect	0.08	0.85
Sleep problems	0.32	0.70

^PCS^Physical HRQL composite score; ^ECS^Emotional HRQL composite score

A set of hierarchical multiple linear regression analyses [31] were performed to investigate whether the initial CD4 value, viral response, adherence and subjective experience of adverse effects predicted the change in HRQL. The two analyses (one for PCS and one for ECS) were conducted in two forced steps with the *follow-up *PCS and ECS as dependent variables. The *pre PI-ART *PCS and ECS, respectively, were entered in the first step, while the three variables viral response, adherence and subjective experience of adverse effects were entered in the second step together with the initial CD4 value.

### Ethical approval

The study was approved by the Local Ethical Committee at Huddinge University Hospital. Information about the study was given to the subjects in connection with an ordinary, scheduled visit to the clinic.

## Results

### Demographic and medical data

At *pre PI-ART*, the mean age of the 72 patients was 41 (SD 9, range 23 – 65) years, 59 were of Swedish origin, 61 had prior antiretroviral treatment with only nucleoside analogue reverse transcriptase inhibitors, 32 had a CD4 count of <200 × 10^6 ^cells l^-1 ^and 28 had an AIDS diagnosis. Further demographic and medical data are shown in Table 2.

**Table 2:** Demographic and medical data regarding a Swedish sample of HIV-infected persons before the start of PI-ART (*n *= 72)

		**No. of subjects**	**Percentage**	
**Mode of transmission**				
Male to male		69	96	
Female to male/male to female		3	4	
**Working status**				
Full or part time work/studies		36	50	
Sick leave or disability pension		31	43	
Not stated		5	7	
**Education**				
Compulsory school		13	18	
Upper secondary school/high school		27	38	
University studies		27	38	
Not stated		5	7	
**Having a partner**:			
Yes/no		30/41	42/57
not stated		1	1

**CD4 count**, cells × 10^6 ^l^-1^:	median			127
	range			1–660
**HIV RNA**, log_10 _copies ml^-1^:	median			4.87
	range			2.70–6.18
**Time since first start of antiretroviral treatment**, months:	mean			33
	SD			31.6

^PI-ART^Protease inhibitor based antiretroviral therapy; ^SD^Standard deviation

### Changes from *pre PI-ART *to *follow-up*

From the *pre PI-ART *to the *follow-up*, the CD4 count of the surviving patients increased from a median (Md) of 150 (range 1 – 660) × 10^6 ^cells l^-1 ^to 325 (range 5 – 840) × 10^6 ^cells l^-1 ^(p < 0.001) and the HIV-1 RNA level decreased from 4.90 (range 2.70 – 6.00) log_10 _copies ml^-1 ^to 2.70 (range 2.70 – 6.31) log_10 _copies ml^-1 ^(p < 0.001). Thirty patients had switched PI during the first two years of PI-ART. A total of 36 subjects were classified as viral responders and 18 as viral non-responders.

#### The patients' emotional well-being deteriorated during the first two years of PI-ART

The *pre PI-ART *and *follow-up *results of the composite scores and the single SWED-QUAL scales are shown in Table 3. The ECS decreased from *pre PI-ART *to *follow-up *(Md 71.2, interquartile range (IQR) 47.1 – 86.9 versus Md 63.6, IQR 41.0 – 77.3; p < 0.01;). A comparison of the single SWED-QUAL scales at *pre PI-ART *with the same scales at *follow-up *revealed statistically significant decreases in the SWED-QUAL scales role limitations due to emotional health (*pre PI-ART *Md 88.9, IQR 66.7 – 100 versus *follow-up *Md 77.8, IQR 50.0 – 100; p < 0.05) and emotional well-being: negative affect (*pre PI-ART *Md 62.5, IQR 37.5 – 94.8 versus *follow-up *Md 50.0, IQR 25.0 – 75.0; p < 0.01; Table 3). The effect sizes of the change were 0.34 for the PCS, 0.36 for role limitations due to emotional health and 0.37 for emotional well-being: negative affect.

**Table 3:** The Swedish Health-Related Quality of Life Questionnaire (SWED-QUAL) results before and after 2 years of protease inhibitor based antiretroviral therapy (*pre PI-ART *and *follow-up *respectively). The higher the score the better the health-related quality of life (n = 54)

	**Score**		
		
	***Pre PI-ART *Median (IQR)**	***Follow-up *Median (IQR)**	***P*-value^a^**
Physical HRQL composite score (PCS)	79.3 (64.5–92.5)	77.2 (58.4–92.9)	NS
Emotional HRQL composite score (ECS)	71.2 (47.1–86.9)	63.6 (41.0–77.3)	<0.01
Physical functioning	95.2 (81.0–95.2)	95.2 (84.5–100)	NS
Mobility	100 (66.7–100)	100 (66.7–100)	NS
Satisfaction with physical ability	66.7 (33.3–83.3)	66.7 (33.3–100)	NS
Pain	100 (53.8–100)	100 (84.7–100)	NS
Role limitations due to physical health	66.7 (55.6–100)	88.9 (50.0–100)	NS
Sexual functioning	75.0 (41.7–91.7)	58.3 (41.7–91.7)	NS
Role limitations due to emotional health	88.9 (66.7–100)	77.8 (50.0–100)	<0.05
Emotional well-being, positive affect	72.9 (47.9–83.3)	62.5 (37.5–83.3)	NS
Emotional well-being, negative affect	62.5 (37.5–94.8)	50.0 (25.0–75.0)	<0.01
Sleep problems	67.9 (39.3–87.5)	57.1 (36.6–75.0)	NS
Satisfaction with family life	72.9 (49.5–90.6)	72.9 (55.7–85.4)	NS
Relation to partner	87.5 (75.0–100)	79.2 (52.1–95.8)	NS
General health perception	55.6 (36.1–75.0)	63.9 (38.9–80.6)	NS

^a^Wilcoxon signed ranks test, change from *pre PI-ART *to *follow-up*; ^NS^Non significant; ^IQR^Interquartile range; ^HRQL^Health-related quality of life

#### Subjective experience of adverse effects contributed to deteriorated emotional HRQL at follow-up

Forty-seven (87 %) of the 54 patients were classified as adherent and seven (13 %) as non-adherent. The mean VAS score for subjective experience of adverse effects at *follow-up *was 3.39 (SD 2.95, Md 2.57, IQR 0.79 – 5.84) cm on a 10 cm scale.

Two hierarchical multiple regression analyses were performed to investigate whether the change in physical and emotional HRQL composite scores could be predicted by initial CD4 count, viral response, adherence and subjective experience of adverse effects (Table 4). Only the ECS model showed a statistically significant R^2 ^change; that is, the subjective experience of adverse effects predicted a decrease in the emotional HRQL.

**Table 4:** Hierarchical multiple linear analyses of the influence of initial CD4 value and the variables viral response, subjective experience of adverse effects and adherence on the change in PCS and ECS from before start (*pre PI-ART*) to after two year of treatment (*follow-up*)

	***Follow-up *PCS**		***Follow-up *ECS**	
	
**Step 1**	**Partial correlation^*a*^**		**Partial correlation^*a*^**	
*Pre PI-ART *PCS	0.722***		-	
*Pre PI-ART *ECS	-		0.681***	
				
R^2^	0.605***		0.456***
F	78.26		42.72
d.f. (regression;residual)	1;51		1;51
	
**Step 2**	**Partial correlation**		**Partial correlation**	

Viral response	0.202		0.076	
Adherence	0.167		0.235	
Adverse effects	0.091		-0.366*	
*Pre PI-ART *CD4 value	-0.043		-0.024	
				
R^2^-change	0.028		0.128*
F-change	0.91		3.63
d.f. (regression;residual)	4;47		4;47

^PCS^Physical HRQL composite score; ^ECS^Emotional HRQL composite score; ^PI-ART^Protease inhibitor based antiretroviral therapy; ^*a*^partial correlation of the final model (step 2); *p < 0.05; ***p < 0.001

### Internal consistency of the SWED-QUAL scales

Cronbach's α reliability estimates for the 11 SWED-QUAL multi-item scales for the *pre PI-ART *and *follow-up *ranged between 0.74 and 0.92 indicating good internal consistency. The internal consistency for the two HRQL composite scores was for the PCS α 0.88 and 0.85 (*pre PI-ART *and *follow-up*, respectively) and for the ECS α 0.91 and 0.89 (*pre PI-ART *and *follow-up*, respectively).

## Discussion

In this study we used the SWED-QUAL instrument to investigate the HRQL of 54 relatively advanced HIV patients before and after two years of first generation PI-ART in a setting where protease inhibitors were introduced. We also studied the patients' viral outcome, self reported adherence and subjective experience of adverse effects and the relationship between these variables and the development in HRQL. To minimise multiple comparisons, a set of linear regression models were used. To increase the power to detect relations as a result of these models, we created two SWED-QUAL composite scores (PCS and ECS) through a data reduction procedure.

The main findings from the present study were that the physical HRQL remained stable while the emotional HRQL deteriorated for two years of first generation PI-ART and that subjective experience of adverse effects was the strongest predictor of the deterioration in emotional HRQL ratings.

The present study thus suggests that first generation PI-ART interrupted the progressive negative development of the physical domain of HRQL that has been reported in studies performed before the introduction of PI-ART [9,10,16,32,33]. Our findings are consistent with those of Goujard *et al. *[34] and Burgoyne *et al. *[18] who also failed to detect any changes in HRQL after one and a half and four years, respectively, when monitoring patients in a period after PI-ART had become available.

The somewhat surprising decrease in emotional health found in the present study has, to our knowledge, not been reported in other longitudinal studies of PI-ART. Longitudinal studies performed before the introduction of PI-ART have, in general, showed stable or improved mental/emotional health over time [9,10,16,35]. We also found stable scores in the emotional domain of HRQL in a previous pre PI-ART era study, where patients with no or only single drug antiretroviral therapy were followed for two years (own unpublished data). However, a study investigating the HRQL of patients receiving didanosine monotherapy or in combination with delavirdine (not approved in Europe due to side effects) showed slightly declining mental health scores for up to two years after the start of the trial [33]. After the introduction of PI-ART, improvements in depressive symptoms and mental health was reported in a shorter time period, i.e. after up to one year following the addition of a PI to existing antiretroviral therapy [36,37]. Similarly, Rabkin *et al. *[38] found a reduction in psychological distress and clinical depression during a two-year period when PI-ART became widely available. In the latter study, however, PI-ART was introduced to the cohort continuously during the follow-up, resulting in a mean time with PI-ART shorter than two years (i.e. the analysis did not evaluate patients before and after start of PI-ART in a consistent manner). From a longer term perspective, however, emotional HRQL was found to be stable over a four-year period [18].

When we further investigated the relation between the change in HRQL and the variables viral response, adherence, subjective experience of adverse effects and baseline CD4 value, our study showed that it was the subjective experience of adverse effects that contributed most to the deterioration in the emotional HRQL. It should be stressed that our measure of adverse effects was a global single item, where the patients were asked to rate their total perception of adverse effects during the previous month and that we did not sub-analyse this experience further into different symptoms. However, the negative impact of perceived side effects/symptoms on HRQL, is confirmed in a great number of quantitative investigations [12,18,37-42]. Also, the results from our quantitative study agreed with those of a qualitative study that aimed to elicit the patients' real-life descriptions of their experience of combination therapy. Erlen & Mellors [43] found that side effects were one of the major problems associated with the therapy. Furthermore, symptoms or adverse events have been shown to be related to medication adherence [44,45]. In order to improve HRQL and adherence, it is therefore crucial to find treatment combinations and strategies that minimise these negative effects and to individualize treatment. The simplified and less toxic treatment regimes available today, with antiretroviral therapy based on three nucleoside analogue reverse transcriptase inhibitors or nucleoside analogues combined with non-nucleoside analogue reverse transcriptase inhibitors or ritonavirboosted PIs once or twice daily [1], may be less liable to have a negative impact on HRQL. This was indicated in a randomised study comparing two nucleoside analogue reverse transcriptase inhibitors combined with either a PI or efavirenz, over a one year period and where the combination with efavirenz had a better influence on HRQL than the combination with the PI [46]. Similarly, Carrieri *et al. *[37] found a positive development of HRQL in patients switching to a non-PI-containing therapy, as compared to patients continuing stable PI-ART. However, these aspects need to be further investigated and there is a need for further study of the effect of different treatment regimes on HRQL, in experienced as well as naïve patients.

Certain study limitations should be emphasized when interpreting the present results. Firstly, the small sample may mean that the study failed to detect further relations between the investigated variables. Secondly, a large proportion of the investigated patients were in an advanced state of their HIV infection and many had previous experience of antiretroviral monotherapy. The inferences drawn from this study may not be applicable to ART naïve patients starting antiretroviral therapy in a less advanced state of the disease. Today treatment is started when certain laboratory criteria are fulfilled and before symptoms, including AIDS-defining disease, are at a high risk of appearing. Thirdly, our study population may not be totally representative of all patients receiving PI-ART. The majority of our patients were well-educated males with a homosexual route of transmission. The influence on HRQL may be different for female patients or patients with other educational status or routes of transmission. It should be noted that we chose to consider viral outcome of the treatment in a longitudinal context, i.e. viral response defined by repeated HIV RNA measures over up to 18 months. The impact on the development of HRQL might have been different if only one single measure of viral load at *follow-up *had been taken into account.

## Conclusion

In this sample of mainly advanced patients, the emotional dimension of HRQL had deteriorated for two years after the start of first generation PI-ART, and the subjective perception of adverse effects made a major contribution to this decrease.

The results of the present study show the importance of studying HRQL in a situation where there is a desperate need for life-saving new therapies. Therefore, consistently taking HRQL into account when treating HIV patients is of the utmost importance. Finding treatment combinations and strategies with the least negative long-term influence on HRQL is essential at a time when those having access to antiretroviral combination therapy have a dramatically increased life span. Furthermore, considering the current numerous treatment options together with the fluctuations in HRQL over time, further short and long-term investigations of HRQL in patients receiving antiretroviral therapy are of the utmost importance.

## Authors' contributions

LEE, ES and GN conceived of the study. All authors made substantial contributions to conception, planning and design. LEE participated in the coordination, carried out the statistical analysis and interpretation of data and drafted the manuscript. GAB participated the coordination and acquisition of the study and helped draft the manuscript. ES participated in the acquisition of the study and helped draft the manuscript. GN participated in the interpretation of data and helped draft the manuscript. All authors read and approved the final manuscript.

## References

[B1] British HIV Association Writing Committee (2003). British HIV Association (BHIVA) guidelines for the treatment of HIV-infected adults with antiretroviral therapy. HIV Med.

[B2] Palella FJ, Delaney KM, Moorman AC, Loveless MO, Fuhrer J, Satten GA, Aschman DJ, Holmberg SD (1998). Declining morbidity and mortality among patients with advanced human immunodeficiency virus infection. HIV Outpatient Study Investigators. N Engl J Med.

[B3] Mocroft A, Katlama C, Johnson AM, Pradier C, Antunes F, Mulcahy F, Chiesi A, Phillips AN, Kirk O, Lundgren JD (2000). AIDS across Europe, 1994–98: the EuroSIDA study. Lancet.

[B4] Safrin S, Grunfeld C (1999). Fat distribution and metabolic changes in patients with HIV infection. AIDS.

[B5] Carr A, Cooper DA (2000). Adverse effects of antiretroviral therapy. Lancet.

[B6] Ragsdale D, Morrow JR (1990). Quality of life as a function of HIV classification. Nurs Res.

[B7] Wu AW, Rubin HR, Mathews WC, Ware JE, Brysk LT, Hardy WD, Bozzette SA, Spector SA, Richman DD (1991). A health status questionnaire using 30 items from the Medical Outcomes Study. Med Care.

[B8] Wachtel T, Piette J, Mor V, Stein M, Fleishman J, Carpenter C (1992). Quality of life in persons with human immunodeficiency virus infection: Measurement by the Medical Outcomes Study Instrument. Ann Intern Med.

[B9] Holzemer WL, Wilson HS (1995). Quality of life and the spectrum of HIV infection. Annu Rev Nurs Res.

[B10] Wu AW, Rubin HR (1994). Approaches to health status assessment in HIV disease overview of the conference. Psychol Health.

[B11] Call SA, Klapow JC, Stewart KE, Westfall AO, Mallinger AP, DeMasi RA, Centor R, Saag MS (2000). Health-related quality of life and virologic outcomes in an HIV clinic. Qual Life Res.

[B12] Lorenz KA, Shapiro MF, Asch SM, Bozzette SA, Hays RD (2001). Associations of symptoms and health-related quality of life: findings from a national study of persons with HIV infection. Ann Intern Med.

[B13] Murri R, Fantoni M, Borgo CD, Visona R, Barracco A, Zambelli A, Testa L, Orchi N, Tozzi V, Bosco O, Wu AW (2003). Determinants of health-related quality of life in HIV-infected patients. AIDS Care.

[B14] Ichikawa M, Natpratan C (2004). Quality of life among people living with HIV/AIDS in northern Thailand: MOS-HIV Health Survey. Qual Life Res.

[B15] Kemmler G, Schmied B, Shetty-Lee A, Zangerle R, Hinterhuber H, Schüssler G, Mumelter B (2003). Quality of life of HIV-infected patients: psychometric properties and validation of the German version of the MQOL-HIV. Qual Life Res.

[B16] de Boer JB, van Dam FSAM, Sprangers MAG (1995). Health-related quality-of-life evaluation in HIV-infected patients. A Review of the literature. Pharmacoeconomics.

[B17] Nieuwkerk PT, Gisolf EH, Reijers MHE, Lange JMA, Danner SA, Sprangers MAG (2001). Long-term quality of life outcomes in three antiretroviral treatment strategies for HIV-1 infection. AIDS.

[B18] Burgoyne RW, Rourke SB, Behrens DM, Salit IE (2004). Long-term quality-of-life outcomes among adults living with HIV in the HAART era: the interplay of changes in clinical factors and symptom profile. AIDS Behav.

[B19] Burgoyne R, Renwick R (2004). Social support and quality of life over time among adults living with HIV in the HAART era. Soc Sci Med.

[B20] Brorsson B, Ifver J, Hays RD (1993). The Swedish Health-Related Quality of Life Survey (SWED-QUAL). Qual Life Res.

[B21] Stewart AL, Hays RD, Ware JE (1988). The MOS Short-form General Health Survey: Reliability and validity in a patient population. Med Care.

[B22] Tarlov AR, Ware JE, Greenfield S, Nelson EC, Perrin E, Zubkoff M (1989). The Medical Outcomes Study. An application of methods for monitoring the results of medical care. JAMA.

[B23] Stewart AL, Sherbourne CD, Hays RD, Wells KB, Nelson EC, Kamberg CJ, Rogers WH, Berry SH, Ware JE, Stewart AL, Ware JE (1992). Summary and discussion of MOS measures. Measuring Functioning and Well-Being The Medical Outcomes Study Approach.

[B24] Ware JE, Sherbourne CD (1992). The MOS 36-Item Short-Form Health Survey (SF-36). I. Conceptual framework and item selection. Med Care.

[B25] Eriksson LE, Nordström G, Berglund T, Sandström E (2000). The health-related quality of life in a Swedish sample of HIV-infected persons. J Adv Nurs.

[B26] Morisky DE, Green LW, Levine DM (1986). Concurrent and predictive validity of a self-reported measure of medication adherence. Med Care.

[B27] (1994). 1994 revised guidelines for the performance of CD4+ T-cell determinations in persons with human immunodeficiency virus (HIV) infections. Centers for Disease Control and Prevention.. MMWR Recom Rep.

[B28] Hejdeman B, Lenkei R, Leandersson A-C, Hultström AL, Wahren B, Sandström E, Bratt G (2001). Clinical and immunological benefits from highly active antiretroviral therapy in spite of limited viral load reduction in HIV type 1 infection. AIDS Res Hum Retroviruses.

[B29] Shott S (1990). Statistics for health professionals.

[B30] Fayers PM, Machin D (2000). Quality of life: assessment, analysis, and interpretation.

[B31] Cohen J, Cohen P (1983). Applied multiple regression/correlation analysis for the behavioral sciences.

[B32] Wu AW, Rubin HR, Mathews WC, Brysk LM, Bozzette SA, Hardy WD, Atkinson JH, Grant I, Spector SA, McCutchan JA, Richman DD (1993). Functional status and well-being in a placebo-controlled trial of zidovudine in early symptomatic HIV infection. J Acquir Immune Defic Syndr.

[B33] Weinfurt KP, Willke RJ, Glick HA, Freimuth WW, Schulman KA (2000). Relationship between CD4 count, viral burden, and quality of life over time in HIV-1-infected patients. Med Care.

[B34] Goujard C, Bernard N, Sohier N, Peyramond D, Lançon F, Chwalow J, Arnould B, Delfraissy J-F (2003). Impact of a patient education program on adherence to HIV medication. J Acquir Immune Defic Syndr.

[B35] Lubeck DP, Fries JF (1992). Changes in quality of life among persons with HIV infection. Qual Life Res.

[B36] Low-Beer S, Chan K, Yip B, Wood W, Montaner JSG, O'Shaughnessy MV, Hogg RS (2000). Depressive symptoms decline among persons on HIV protease inhibitors. J Acquir Immune Defic Syndr.

[B37] Carrieri P, Spire B, Duran S, Katlama C, Peyramond D, Francois C, Chene G, Lang JM, Moatti JP, Leport C (2003). Health-related quality of life after 1 year of highly active antiretroviral therapy. J Acquir Immune Defic Syndr.

[B38] Rabkin J, Ferrando S, Lin S, Sewell M, McElhiney M (2000). Psychological effect of HAART: a 2-year study. Psychosom Med.

[B39] Blanch J, Rousaud A, Martinez E, Lazzari ED, Milinkovic A, Peri J-M, Blanco J-L, Jaen J, Navarro V, Massana G, Gatell J-M (2004). Factors associated with severe impact of lipodystrophy on the quality of life of patients infected with HIV-1. Clin Infect Dis.

[B40] Hudson A, Kirksey K, Holzemer W (2004). The influence of symptoms on quality of life among HIV-infected women. West J Nurs Res.

[B41] Préau M, Leport C, Salmon-Ceron D, Carrieri P, Portier H, Chene G, Spire B, Choutet P, Raffi F, Morin M, APROCO Study Group (2004). Health-related quality of life and patient-provider relationships in HIV-infected patients during the first three years after starting PI-containing antiretroviral treatment. AIDS Care.

[B42] Tramarin A, Parise N, Campostrini S, Yin DD, Postma MJ, Lyu R, Grisetti R, Capetti A, Cattelan AM, Di Toro MT, Mastroianni A, Pignattari E, Mondardini V, Calleri G, Raise E, Starace F, Palladio Study Group (2004). Association between diarrhea and quality of life in HIV-infected patients receiving highly active antiretroviral therapy. Qual Life Res.

[B43] Erlen JA, Mellors MP (1999). Adherence to combination therapy in persons living with HIV: balancing the hardships and the blessings. J Assoc Nurses AIDS Care.

[B44] Holzemer WL, Corless IB, Nokes KM, Turner JG, Brown MA, Powell-Cope GM, Inouye J, Henry SB, Nicholas PK, Portillo CJ (1999). Predictors of self-reported adherence in persons living with HIV disease. AIDS Patient Care STDS.

[B45] Stone VE, Jordan J, Tolson J, Miller R, Pilon T (2004). Perspectives on adherence and simplicity for HIV-infected patients on antiretroviral therapy (HAART) regimens in predicting adherence. J Acquir Immune Defic Syndr.

[B46] Fumaz CR, Tuldrà A, Ferrer MJ, Paredes R, Bonjoch A, Jou J, Negredo E, Romeu J, Sirera G, Tural C, Clotet B (2002). Quality of life, emotional status, and adherence of HIV-1-infected patients treated with efavirenz versus protease inhibitor-containing regimens. J Acquir Immune Defic Syndr.

